# Real-Time Measurement of Melanoma Cell-Mediated Human Brain Endothelial Barrier Disruption Using Electric Cell-Substrate Impedance Sensing Technology

**DOI:** 10.3390/bios9020056

**Published:** 2019-04-15

**Authors:** Akshata Anchan, Panagiota Kalogirou-Baldwin, Rebecca Johnson, Dan T Kho, Wayne Joseph, James Hucklesby, Graeme J Finlay, Simon J O’Carroll, Catherine E Angel, E Scott Graham

**Affiliations:** 1 Department of Molecular Medicine and Pathology, School of Medical Sciences, Faculty of Medical and Health Sciences, University of Auckland, Auckland 1023, New Zealand; a.anchan@auckland.ac.nz (A.A.); pkalogiroubaldwin@gmail.com (P.K.-B.); rebecca.johnson@auckland.ac.nz (R.J.); dkho024@aucklanduni.ac.nz (D.T.K.); w.joseph@auckland.ac.nz (W.J.); james.hucklesby@auckland.ac.nz (J.H.); g.finlay@auckland.ac.nz (G.J.F.); 2 Centre for Brain Research, University of Auckland, Auckland 1023, New Zealand; s.ocarroll@auckland.ac.nz; 3 Auckland Cancer Society Research Centre, University of Auckland, Auckland 1023, New Zealand; 4 School of Biological Sciences, Faculty of Science, University of Auckland, Auckland 1010, New Zealand; c.angel@auckland.ac.nz; 5 Department of Anatomy and Medical Imaging, School of Medical Sciences, Faculty of Medical and Health Sciences, University of Auckland, Auckland 1023, New Zealand; 6 Maurice Wilkins Centre for Molecular Biodiscovery, University of Auckland, Auckland 1010, New Zealand

**Keywords:** ECIS, impedance, barrier function, endothelium, blood–brain barrier

## Abstract

Electric cell-substrate impedance sensing (ECIS) is an impedance-based method for monitoring changes in cell behaviour in real-time. In this paper, we highlight the importance of ECIS in measuring the kinetics of human melanoma cell invasion across human brain endothelium. ECIS data can be mathematically modelled to assess which component of the endothelial paracellular and basolateral barriers is being affected and when. Our results reveal that a range of human melanoma cells can mediate disruption of human brain endothelium, primarily involving the paracellular route, as demonstrated by ECIS. The sensitivity of ECIS also reveals that the paracellular barrier weakens within 30–60 min of the melanoma cells being added to the apical face of the endothelial cells. Imaging reveals pronounced localisation of the melanoma cells at the paracellular junctions consistent with paracellular migration. Time-lapse imaging further reveals junctional opening and disruption of the endothelial monolayer by the invasive melanoma cells all within several hours. We suggest that the ability of ECIS to resolve changes to barrier integrity in real time, and to determine the route of migration, provides a powerful tool for future studies investigating the key molecules involved in the invasive process of cancer cells.

## 1. Introduction

Malignant melanoma is an aggressive cancer with high propensity to metastasize to the brain [1]. However, metastasis in the brain is relatively difficult and complex due to the presence of the physical structure known as the blood–brain barrier (BBB). The fundamental role of the BBB is to restrict the passage of blood-borne inflammatory molecules and pathogens into the brain, so as to protect the brain from injury. Brain endothelial cells are a key component of the BBB. They are the first cell type with which circulating molecules (or melanoma cells) come into contact with, and so form the first line of defence against potentially injurious blood-borne agents. Therefore, we investigated the barrier integrity of brain endothelial cells when testing melanoma cell invasion and migration (extravasation).

### 1.1. The Structural Composition of the Blood–Brain Barrier

Brain endothelial cells, which make up the vascular wall, structurally form a monolayer of cells, laterally attached to each other by tight junction (TJs) and adherens junctions (AJs). These junctions are formed by a complex repertoire of molecules that maintain cellular structure and junctional stability. Metastasis to the brain would therefore require melanoma cells to sequentially break through protein complexes, traverse endothelial layers, travel into the brain parenchyma and form secondary tumours. This form of migration is called paracellular migration. The detailed mechanism of melanoma migration through the BBB remains mostly unknown, but the process is proposed to occur in multiple ways. This paper focuses on the paracellular extravasation of melanoma cells through the brain endothelial cells’ junctional barrier which differs from the trans-cellular route, the movement of cancer cells through the endothelial cells instead of between them.

### 1.2. Current Understanding of Paracellular Melanoma Metastasis into the Brain

Following breast and lung cancer, melanoma is the third most likely cancer to metastasise to the brain. Clinical studies show that 10 to 50% of patients with advanced melanoma develop secondary neoplasms in the central nervous system (CNS) [2,3] and this is associated with poor prognosis (median survival is approximately six months [1]).

Although intravascular growth and vascular rupture is one suggested pathway of metastasis, it is also hypothesized that cancer cells need to overcome the junctional complexes at the BBB, mimicking what occurs during paracellular migration of leukocytes through the barrier during healing [4] or during inflammation-mediated CNS disorders such as multiple sclerosis (MS). Most mechanistic studies have assessed the metastatic potential of cancer cell lines on endothelial cells, obtained from a variety of tissue sources, through in vitro trans-migratory experiments and overall impedance sensing [5,6,7].

### 1.3. The Utility of Electric Cell-Substrate Impedance Sensing (ECIS)

**Figure 1:**
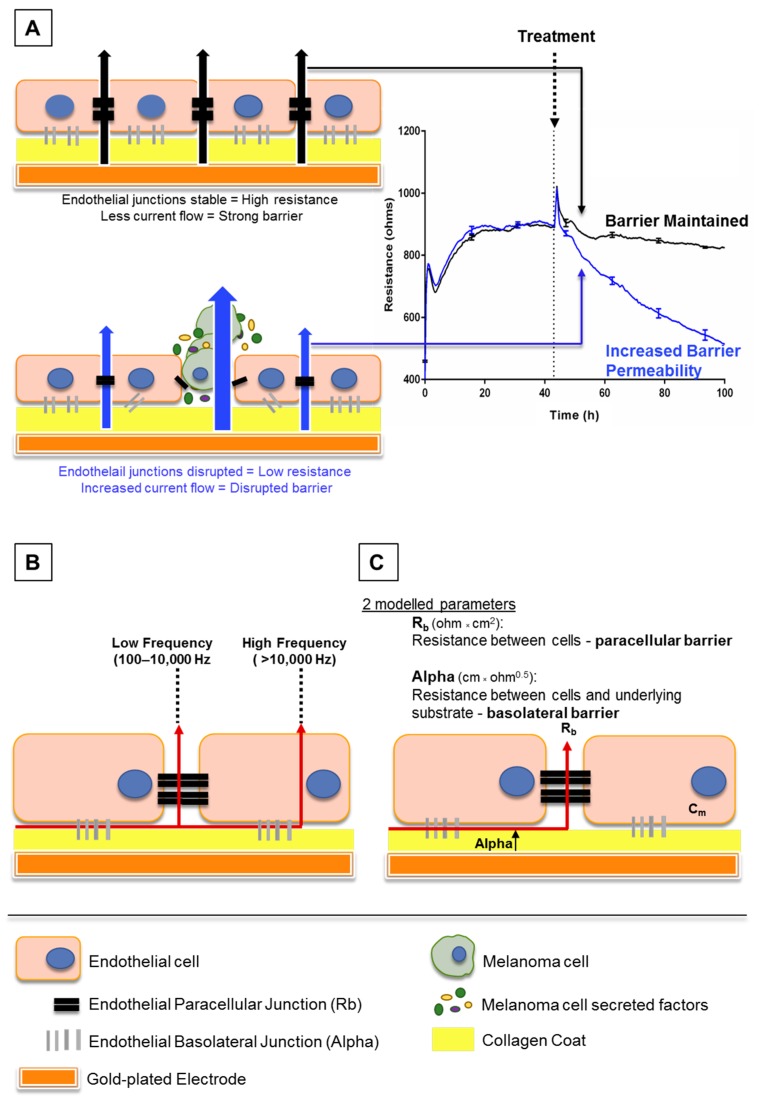
Schematic explaining electric cell-substrate impedance sensing (ECIS) theory. (**A**) This shows endothelial cells growing on gold electrodes which are coated with rat tail collagen I. The endothelial cells form a monolayer by forming junctions with each other and the collagen substrate, shown by black and grey bars respectively. Once these junctions are formed, less current, supplied by the electrode, can flow through the cell junctions. This increases the electrical resistance as seen on the graph (right). As the cells become confluent, maximum resistance is achieved and the resistance plateaus. Upon treatment with factors that disrupt the endothelial barrier, the junctions weaken, current flows more freely between the cells and the electrical resistance decreases (blue trace, right). (**B)** This shows how the ECIS data can be modelled depending on the frequency at which the current is applied by the electrodes. At low frequencies (100–10,000 Hz), current flows only between cells, whereas at high frequencies (>10,000 Hz), the current can also flow through cells. (**C**) Collectively, the low and high frequency data can be modelled to provide information on the resistance between cells (Rb) and resistance between cells and the underlying substrate (Alpha).

Electric cell-substrate impedance sensing or ECIS is a real-time and label-free, impedance-based method. It allows the assessment of various attributes of cell behaviour such as cell adhesion and barrier strength by measuring the impedance across a confluent cell monolayer. Brain endothelial cells can be grown on gold-plated electrodes which apply an AC current through the cells. The potential created can be used to calculate the impedance. This impedance typically increases and then plateaus as the endothelial cells become confluent and form a barrier, restricting current flow. Treating the endothelial cells with various stimuli can then alter the impedance obtained. This may occur if a given stimulus disrupts the barrier integrity, creating a path of least resistance to allow easier current flow (increasing permeability as shown in Figure 1A). With this method, a decrease in impedance or “barrier resistance” suggests loss of barrier strength, as current can freely flow through the disrupted cell monolayer. The ECIS electrodes can measure acute temporal changes in barrier resistance across the endothelial cell layer, and then model the collected data to provide information on separate components of the endothelial cell barrier: paracellular and basolateral (Figure 1C) resistance (described in [8]).

### 1.4. Research Aims

The research aims were to investigate the ability of human melanoma cells to affect barrier strength of human brain-derived endothelial cells, by using a sensitive measure of electrical resistance in an in vitro model of brain endothelium. The research hypothesis was that melanoma cells would disrupt the blood–endothelial barrier.

## 2. Materials and Methods

### 2.1. Cell Culture

#### 2.1.1. Human Cerebral Microvascular Endothelial Cells (hCMVECs)

The human brain endothelial cells (hCMVECs) were purchased from Applied Biological Materials Inc. (ABM, cat# T0259, Richmond, BC, CANADA) and cultured in T75 flasks with M199 growth medium containing 10% FBS, 1 µg/mL hydrocortisone, 3 ng/mL hFGF, 1 ng/mL hEGF, 10 µg/mL heparin, 2 mM GlutaMAX and 80 µM dibutyryl-cAMP (as detailed in Appendix A, Table A1). All culture-ware was coated with 1 µg/cm^2^ collagen I dissolved in 0.02 M acetic acid.

#### 2.1.2. New Zealand Melanoma (NZM) Cells

Malignant melanoma cells (New Zealand Melanoma) were developed from metastatic melanomas in the Auckland Cancer Society Research Centre (ACSRC), described in [9]. Eight metastatic melanoma cell lines (called NZM^*x*^), detailed in Table A2, were used. The cells were cultured with Minimum Essential Medium α (αMEM) containing 5% FBS, 5 μg/mL insulin, 5 μg/mL transferrin and 5 ng/mL sodium selenite (as detailed in Appendix A Table A1). As cancer cells may acquire genetic abnormalities upon multiple replications, none of the NZM lines were used past passage 30.

#### 2.1.3. Peripheral Blood Mononuclear Cells (PBMCs)

Whole blood was collected from healthy donors following informed consent. Ethics approval for blood collection was granted by the University of Auckland Human Participants Ethics Committee (UAHPEC, UoA # 014035). Peripheral blood mononuclear cells (PBMCs) were separated from whole blood using Ficoll-Hypaque (cat# 10771, Sigma Aldrich, St. Louis, MO, USA) density gradient centrifugation with Leucosep tubes (cat# 227289, Greiner Bio-One, Kremsmünster, Austria) as per manufacturer’s protocol. Stocks were frozen at −80 °C. Cells were thawed and used instantly for experiments in RPMI 1640 medium (cat# 11875093, Gibco, Thermo Fisher Scientific, Waltham, MA, USA) supplemented with 10% FBS.

### 2.2. Electrical Cell-Substrate Impedance Sensing (ECIS)

#### 2.2.1. ECIS Setup

The ECIS Zθ system uses 96-well plates lined with interdigitating fingers of gold-plated electrodes (96W20idf), which were coated with 10 mM cysteine for 15 min to maintain the electrode capacitance. The wells were coated with 1 µg/cm^2^ of collagen I dissolved in 0.02 M acetic acid as per cell culture protocol described above. The hCMVECs were seeded at 20,000 cells per well, in 100 µL of M199 growth medium supplemented with 10% FBS. Cells were allowed to proliferate and form high resistance (measured by increase in ohms) monolayers for approximately 48 h. The ECIS machine was run continuously at multi-frequency, so the ECIS system may record resistance across low and high frequencies and then model the recorded resistance into separate components as developed by Giaever and Keese [10] (Figure 1B,C).

ECIS results are presented as the change in overall endothelial barrier resistance, which shows the unmodelled resistance (ohms) over time of each well from the 96W20idf plates. These results are taken from low frequency (at 4000 Hz) data, where the current flows between the endothelial cell–cell junctions. As the system records resistance at several frequencies, the ECIS system can also mathematically model this into the Resistance beta (Rb) or the paracellular resistance (ohm × cm^2^), which represents the most valuable information for endothelial TJ and AJ disruption in the lateral borders of the cell. It also models the data to present the Resistance alpha (Alpha) or basolateral resistance (cm × ohm^0.5^), which gives additional information on endothelial adhesion to the underlying supportive substrate scaffold (i.e., collagen). These results are presented as the modelled resistance, and together form the original overall endothelial barrier resistance (ohms).

#### 2.2.2. Addition of Cells onto the hCMVECs

The NZM cells representing several independent lines were harvested with Versene (1x, Gibco, 15040-066) to protect any cell-surface molecules that aid melanoma cell adhesion. The melanoma cells were then added to the hCMVECs in ECIS plates at three different effector-target (ET) ratios—1 melanoma cell:1 hCMVEC (1:1), 1 melanoma cell:10 hCMVECs (1:10) and 1 melanoma cell:100 hCMVECs (1:100)—in 100 µL of complete αMEM medium per well. Medium-only control was 100 µl of complete αMEM added to the hCMVECs. PBMCs were added at four different ET ratios (10:1, 1:1, 1:10, 1:100) in 100 µL of complete RPMI medium (100 µl of complete RPMI was added to the hCMVECs as medium-only control).

#### 2.2.3. Addition of Melanoma-Conditioned Medium onto the hCMVECs

NZM cells were each seeded at 900,000 cells in a T75 flask. Medium was collected from the cells from Day 1 (day of cell passage) to Day 7 (90% confluency) on alternating days. The collected conditioned medium (CM) was centrifuged for 10 min at 300× g to remove cellular debris and the supernatant was stored separately at –80 °C. On the day of treatment, 100 µl of melanoma-conditioned medium was pre-warmed and added to 20,000 hCMVECs per well on the ECIS plate, which had been grown for ~48 h until they had formed a continuous monolayer of high resistance, sustained through their paracellular barrier (Rb). Medium-only control (100 µl of αMEM) was added to the hCMVECs. All ECIS data were analysed using GraphPad Prism (version 7.03, GraphPad, San Diego, CA, USA) software and plotted as the mean (± SD) of multiple replicates (minimum of three).

### 2.3. Immunocytochemistry

The hCMVECs were plated in collagen-coated 96-well plates (cat# 161093, Invitrogen, Carlsbad, CA, USA) and allowed to grow for 48 h, after which CellTracker™ Green CMFDA (5-chloromethylfluorescein diacetate, cat# C7025, Invitrogen)-stained NZM cells (1 µM) were added at a 1:10 ratio. Cells were incubated in co-culture for 30 min. Fixation and permeabilisation were performed with 4% paraformaldehyde and 0.1% Triton X in PBS, respectively. Blocking was performed using 1% bovine serum albumin (BSA). Cells were stained for α-catenin (dilution 1:100, cat# 13-9700, Invitrogen).

### 2.4. Live-Cell Imaging

Melanoma cell invasion was imaged using the Nikon BioStation (Nikon Instruments Inc., Tokyo, Japan). Brain endothelial cells (400,000) were seeded in a 35 mm Petri dish (Nunc 153006, Roskilde, Denmark) in 1.5 mls of M199 growth medium. Cells were cultured as per growth conditions described above, for 48 h to achieve confluency. The brain endothelial cells were then stained with 1 µM CellTracker™ Green CMFDA (Thermo Fisher Scientific, Waltham, MA, USA) and imaged for 2 h, after which 40,000 melanoma cells were added in 500 µL of complete αMEM and imaged for a further 5 h. The software was programmed to acquire images (phase and GFP channel) every 5 min across multiple sites throughout the entire time-lapse experiment.

### 2.5. Statistics

RStudio (version 1.1.414, RStudio, Inc., Boston, MA, USA) was used to conduct two-way analysis of variance followed by Tukey’s range test. All probabilities shown are relative to a media only control at the final time point. Normality was confirmed using both visual inspection of the data and the Shapiro–Wilk test of normality. All graphs were generated using GraphPad Prism 7 (La Jolla, CA, USA).

## 3. Results

### 3.1. Decrease in Brain Endothelial Barrier Strength upon Addition of Melanoma Cells

**Figure 2:**
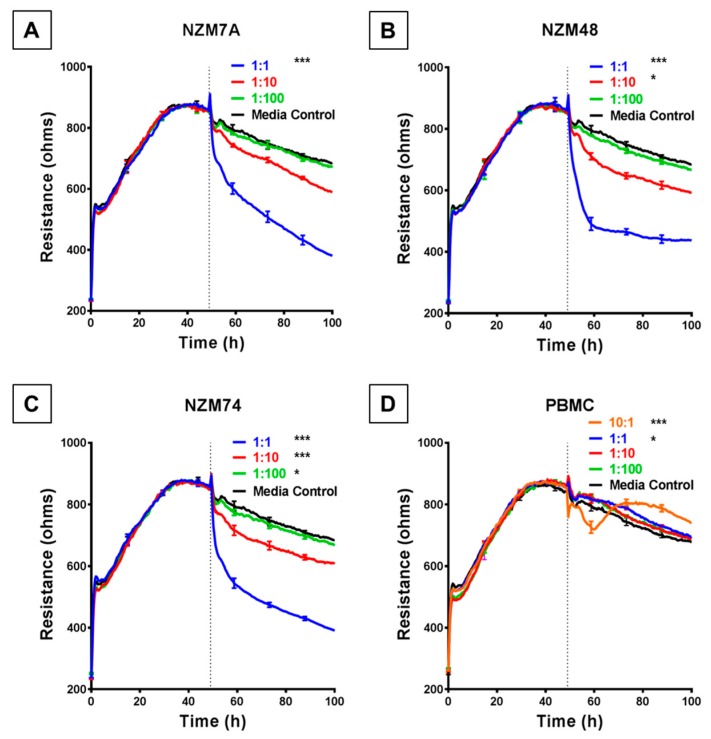
ECIS reveals decreases in hCMVEC barrier resistance following exposure to NZM melanoma cells. (**A**–**C**) Unmodelled resistance (at 4000 Hz) of hCMVECs over time after addition of three different NZM cell lines. Cells were added at different effector:target (E:T) ratios, where 1:1 shows 1 NZM cell added to 1 endothelial cell. Data shown as mean ± SD (*n* = 3 wells) from one experiment which is representative of four independent experiments. (**D**) Unmodelled resistance (at 4000 Hz) of hCMVECs over time after addition of PBMCs as a cell-control, added at different effector:target (E:T) ratios, where 10:1 shows 10 PBMCs added to 1 endothelial cell. RPMI with 10% FBS was used as medium-only control. hCMVECs were seeded at 20,000 cells per well. PBMCs/NZM cells were added at 48 h (dotted line). Data shown as mean ± SD (*n* = 3 wells) from a single experiment; endpoints from three independent experiments were compared relative to their media control using two-way ANOVA with Tukey’s range test (* *p* < 0.05, *** *p* < 0.001).

We used the ECIS system to detect, calculate and record barrier resistance across the endothelial monolayer and assess any changes in real time to the resistance upon addition of melanoma cells. Each human melanoma line decreased the total barrier resistance (measured at 4000 Hz) compared to the medium-only control. Notably, for each melanoma line, this was observed to occur in a cell number-dependent manner (Figure 2), for which a higher ET ratio (higher melanoma cell number to endothelial cell) was associated with a greater loss of barrier resistance of the brain endothelial cells. This decrease in barrier resistance was rapid, occurring within the first 30–60 min of melanoma cell addition and was maintained for the duration of the experiment.

This substantial decrease in endothelial barrier resistance was not observed following addition of human PBMCs, which were used as the cellular control. When the PBMCs were added at an extremely high ET ratio of 10:1 (Figure 2D), there was an acute and transient reduction in barrier resistance. However, after a period of 8–10 h the barrier strength increased, indicating the reformation of the barrier.

### 3.2. Modelled Decrease in Brain Endothelial Barrier Strength upon Addition of Melanoma Cells

**Figure 3:**
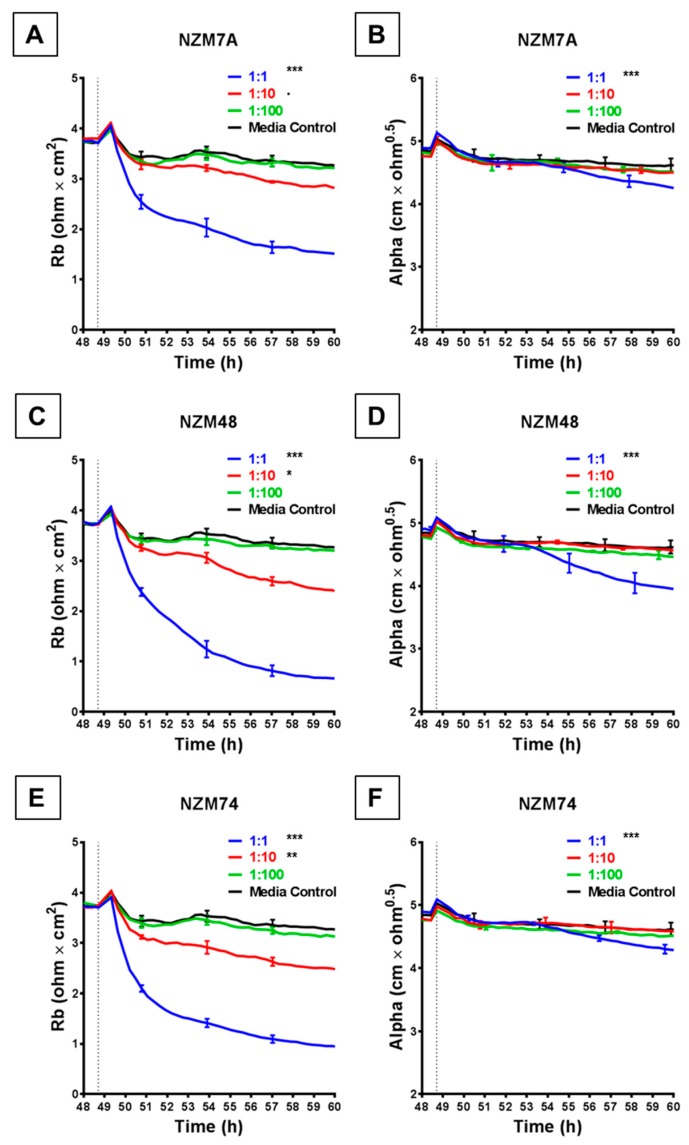
ECIS reveals decreases in hCMVEC paracellular and basolateral resistance following exposure to melanoma cells NZM7, 48 and 74. (**A**), (**C**), (**E**) Modelled paracellular resistance (Rb) of hCMVECs over time after addition of NZM cell lines. (**B**), (**D**), (**F**) Modelled basolateral resistance (Alpha) of hCMVECs over time after addition of NZM cell lines. NZM cells were added at 48 h (dotted line). Data shown as mean ± SD (*n* = 3 wells) from a single experiment; endpoints from three independent experiments were compared relative to their media control using two-way ANOVA with Tukey’s range test (*p* < 0.1, * *p* < 0.05, ** *p* < 0.01, *** *p* < 0.001).

The rapid and substantial loss of barrier resistance can potentially be explained by various distinct mechanisms, which cannot be distinguished by changes in overall resistance. We hypothesise that these mechanisms may involve melanoma cells (i) killing the endothelial cells or (ii) inducing disassembly at the paracellular junctions. The ECIS technology is unique in that the multi-frequency impedance data can be mathematically modelled to resolve changes in resistance into contributions from basolateral focal adhesion (referred to as Alpha) and paracellular adhesion (Rb). This analysis is shown in Figure 3 and reveals that it is the paracellular barrier resistance that decreases substantially after addition of all three melanoma lines assessed. As with the overall resistance, this loss of paracellular barrier resistance was fast, occurring within the first 30–60 min with NZM7, NZM48 and NZM74 (Figure 3A,C,E), showing that the paracellular and overall barrier resistance shared similar profiles of endothelial barrier disruption. Paracellular barrier disruption was also cell number-dependant, as with overall resistance. Note that there were detectable paracellular (Rb) changes with each of the melanoma lines even at an ET ratio of 1 melanoma cell per 10 endothelial cells, highlighting the sensitivity of detection.

Each melanoma line also decreased the basolateral resistance at an ET ratio of 1:1 but not at the lower ET ratios. An important observation from the modelled data demonstrates that for these lines, the Alpha parameter (focal adhesion) did not change substantially for at least 2 or 3 h after melanoma addition (Figure 3B,D,F). This is important, as it indicates that the predominant and earliest change to the overall resistance is due to changes in the paracellular resistance and that the melanoma cells affect the paracellular barrier first, followed by the focal adhesion, which is what would be expected during paracellular migration.

**Figure 4:**
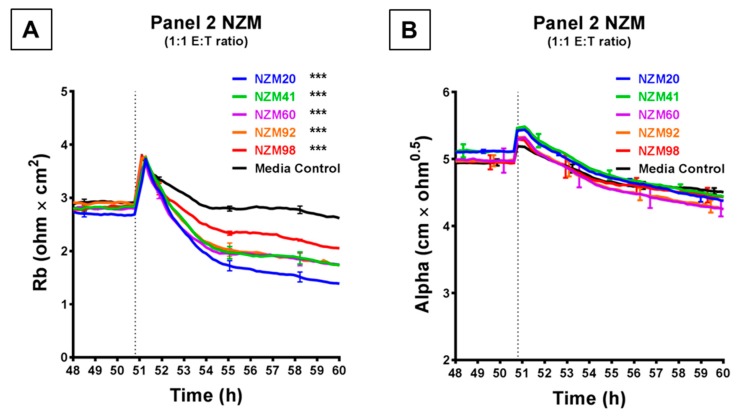
ECIS demonstrates that while Panel 2 NZM melanoma cells induce a decrease in hCMVEC paracellular resistance, they do not influence basolateral resistance. Modelled (**A**) paracellular resistance and (**B**) basolateral resistance of hCMVECs over time after addition of Panel 2 NZM melanoma cell lines. Cells were added at a 1:1 E:T ratio. hCMVECs were seeded at 20,000 cells per well. NZM cells were added at 48 h (dotted line). Data shown as mean ± SD (*n* = 3 wells) from a single experiment; endpoints from two independent experiments were compared relative to their media control using two-way ANOVA with Tukey’s range test (*** *p* < 0.001).

Figure 4 shows the comparison of a further five melanoma lines, which were each assessed at an ET ratio of 1:1 (the ratio at which the largest decrease in barrier resistance was previously achieved) for their ability to disrupt the endothelial barrier on ECIS. As shown in Figure 4A, each of these melanoma lines decreased the paracellular barrier resistance to varying extents, with NZM20 causing approximately 50% decrease in Rb, within 10 h. NZM98 however was the least effective and decreased the paracellular barrier resistance only by about 20% maximally. Interestingly, (unlike in Figure 3) the basolateral barrier resistance for all five cell types remained unchanged on ECIS for up to 10 h post melanoma cell addition (Figure 4B). The differences in time and magnitude suggest potentially different molecular mechanisms of barrier breach, but each implicates the paracellular route due to the predominant effect on Rb.

### 3.3. Decrease in Brain Endothelial Barrier Strength upon Addition of Melanoma Cell-Conditioned Medium

**Figure 5:**
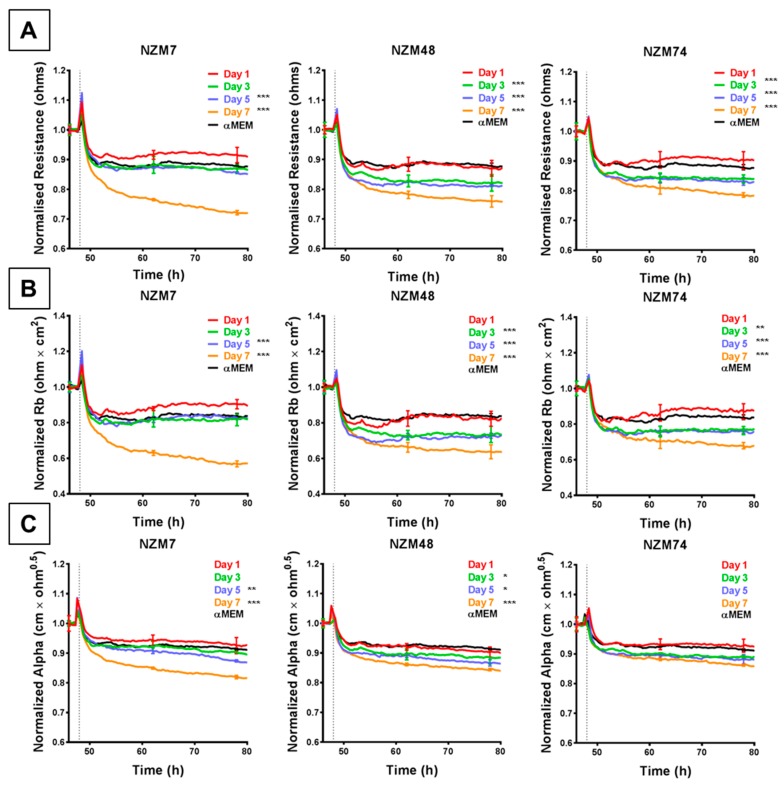
ECIS reveals that melanoma cell (NZM7, 48 and 74)-conditioned medium (CM) disrupts hCMVEC paracellular and basolateral resistance. (**A**) Unmodelled resistance (at 4000 Hz) of hCMVECs over time after addition of CM from NZM cell lines. Modelled (**B**) paracellular resistance (Rb) and (**C**) basolateral resistance (Alpha) of hCMVECs over time after addition of CM from NZM cell lines. NZM CM was added at 48 h (dotted line). αMEM is medium-only control. Data shown as mean ± SD (*n* = 3 wells) from a single experiment; Endpoints from three independent experiments were compared relative to their media control using two-way ANOVA with Tukey’s range test (* *p* < 0.05, ** *p* < 0.01, *** *p* < 0.001).

Conditioned medium (CM) was collected from melanoma cells (NZM7, NZM48, NZM74) over a duration of seven days to specifically assess whether the cells released soluble factors (e.g., cytokines) that affect the endothelial barrier strength. The melanoma CM decreased endothelial barrier resistance compared to medium-only control, but to a lesser extent than the intact cells (~ 20% compared to up to 70% with cells). This was evident in their change in overall barrier resistance (Figure 5A) as well as the paracellular (Figure 5B) and basolateral (Figure 5C) resistance. As with the cells, the paracellular resistance decreased more than the basolateral resistance (20% to 10% respectively). However, temporally these decreases occurred at the same time. Notably, CM collected from later days induced a greater decrease in barrier resistance than CM collected from earlier days, where CM from Day 7 showed the greatest change in barrier resistance for each melanoma line.

### 3.4. Melanoma Cells Rapidly Localise to the Junctional Space of Human Microvascular Endothelial Cells

**Figure 6:**
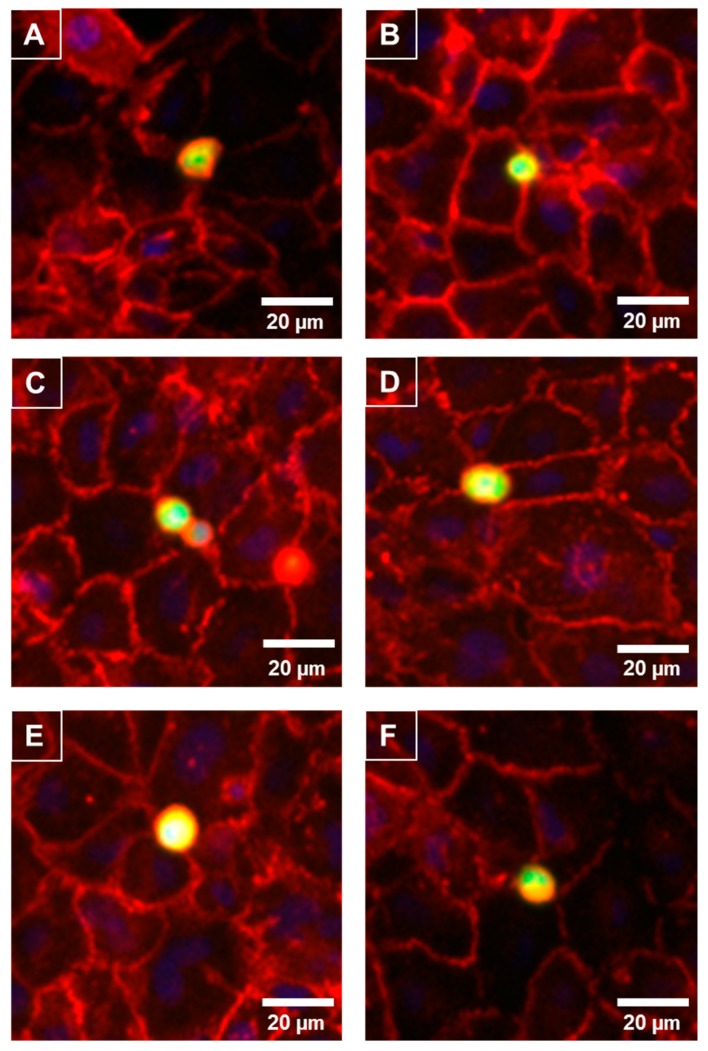
Preferential adhesion of melanoma cells (NZM7) to hCMVEC endothelial junctions. NZM7 cells were live-stained with CMFDA (green) and applied to the apical face of hCMVECs. Cells were co-cultured for 30 min, fixed and endothelial junctions were visualized with anti-α-catenin (red). Nuclei were visualized using DAPI. (**A**–**F**) Multiple examples of a melanoma cell (green/yellow) localising at endothelial junctions (red). Images (**A**) and (**D**) are good examples of the melanoma cell residing at a multi-junctional location (corner of several endothelial cells). (**B**) shows a small melanoma cells at the junction of a large clearly visible endothelial cell. (**C**) is an example of multiple melanoma cells adhered at the same junctional positon. (**E**) exemplifies a large melanoma cell positioned at multiple endothelial junctions and (**F**) shows a melanoma cells on the junctional border of four neighbouring endothelial cells. Images shown are representative of two independent experiments. Scale bar is 20 µm.

As the melanoma cells affected the paracellular (Rb) barrier strength so quickly and predominately, it was predicted that the melanoma cells would reside at the paracellular junctions shortly after their addition. The melanoma cells were live-labelled with CMFDA prior to addition and fixed 30 min after addition. The location of the paracellular junctions was revealed using α-catenin localisation (red, Figure 6), which is expressed highly and uniformly in these brain endothelial cells. Figure 6 revealed that within 30 min of addition the melanoma cells (green) presented directly above the paracellular junctions. This confirmed the temporal ECIS data predicting that the locus of barrier disruption effect was paracellular rather than trans-cellular or through the cell body. Some melanoma cells resided directly over junctional spaces corresponding to up to three endothelial cells. There was also evidence of melanoma cells aligning with endothelial junctional borders in clusters.

### 3.5. Time-Lapse of Melanoma and Brain Endothelial Cell Co-Culture

**Figure 7:**
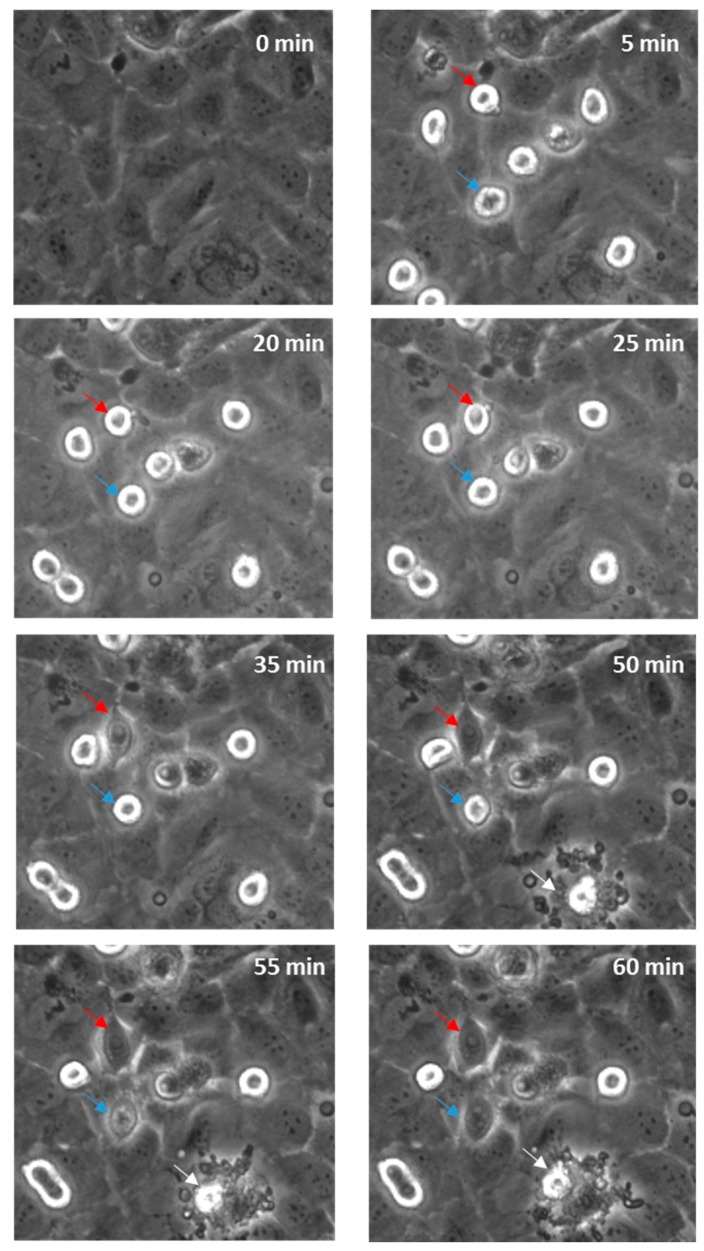
Time-lapse series illustrating melanoma cells (NZM7) adhering to and integrating into hCMVEC endothelial layer. Time 0 represents the time point immediately before the addition of the melanoma cells, which appear spherical and refractile. The red and blue arrows follow spherical melanoma cells above endothelium that distinctly integrate into the endothelial monolayer over time. White arrow indicates evidence of endothelial cell death. Images shown are representative of two independent experiments.

Real-time live-cell imaging was performed next to confirm that the disruption was paracellular as predicted. Figure 7 shows this with bright field imaging where the melanoma cells, visible as refractile, spherical cells, are highlighted with arrows. Within 60 min, several cells markedly changed their morphology and appeared to integrate with the endothelial monolayer. See also supplemental data in Supplementary Materials, Video S1 (AVI movie for the data in Figure 7). There was also evidence of the occasional (albeit rare; not evident in all areas analysed) event of an endothelial cell dying, which was in close proximity to a melanoma cell. It is however challenging to ascertain whether the melanoma cells have invaded the endothelial layer or have simply spread out across the endothelial surface, using this form of imaging. Therefore, the endothelial cells were counterstained with CMFDA to demarcate the endothelial cytoplasm. Unlabelled melanoma cells were then added and imaged autonomously every 5 min using a Nikon BioStation platform (live-cell imaging system).

### 3.6. Melanoma-Mediated Retraction of Brain Endothelial Cells in Co-Culture

**Figure 8:**
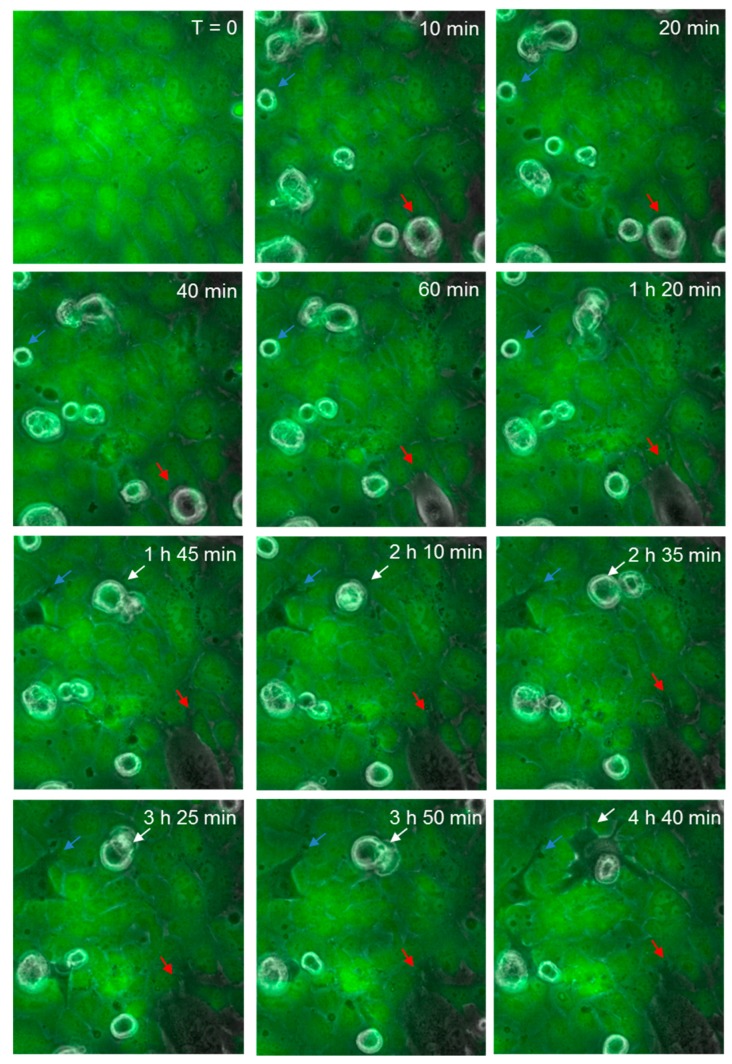
Time-lapse series illustrating melanoma cells (NZM74) integrating into hCMVEC endothelial layer. Images show endothelial cells stained with CMFDA (green), merged with phase only to visualize unstained spherical melanoma cells. Coloured arrows indicate melanoma cells that extend protrusions between endothelial cells, which over time enable them to penetrate the endothelial monolayer. Images shown are representative of two independent experiments.

Figure 8 shows the continuous monolayer of the endothelial cells (green), attached at cell to cell junctions, with CMFDA staining throughout the cell body. As before, spherical melanoma cells are clearly visible. Over time (60–180 min), several melanoma cells were observed to squeeze between the green endothelial cells and continuously push them aside. These were clearly integrated within the endothelial cell layer and melanoma cytoplasmic processes were observed to push their way between neighbouring cells. These images (Figure 7 and Figure 8) clearly indicate that the major mechanism of disruption as indicated by ECIS is via the paracellular route and that melanoma-mediated killing may happen but to a very minor extent. These results were seen for three melanoma cell lines tested (NZM7, NZM48 and NZM74) of which NZM7 (Figure 7) and NZM74 (Figure 8) are shown. See also supplemental data in Supplementary Materials, Video S2 (AVI movie for the data in Figure 8).

## 4. Discussion

**Figure 9:**
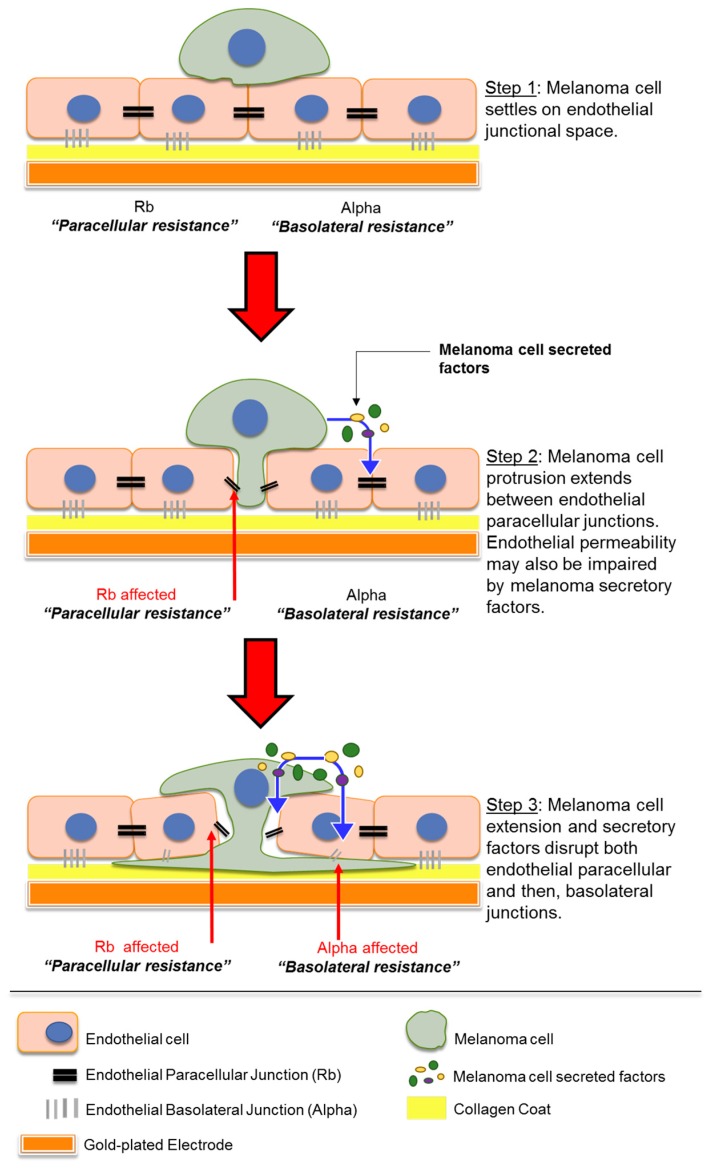
Schematic showing possible sequence of events which occur when melanoma cells encounter endothelial monolayer. Paracellular resistance (Rb) and basolateral resistance (Alpha) together form the overall barrier resistance of the endothelial monolayer. Diagram not to scale.

The aggressiveness of melanoma stems from its ability to metastasize to various organs in the body. Melanoma particularly has a high propensity to metastasize to the brain [1], leading to a devastating prognosis after secondary tumour formation. The mechanisms behind melanoma metastasis are of great interest and although various studies state the importance of several molecules, the complete process of melanoma migration through the human blood–brain barrier (BBB) is not fully understood. In this paper, we specifically investigated the ability of ECIS technology to monitor and measure the temporal kinetics of melanoma cell-mediated disruption in an in vitro model of the human blood–brain barrier endothelium. The key findings are (i) the importance of conducting real-time assessment of melanoma cells on endothelial barrier function due to the fast nature of barrier disruption, (ii) the ability of ECIS data to be modelled to predict effects on the paracellular junctions and basolateral adhesion (Figure 9), (iii) the relative sensitivity of the technology to use low ET ratios of invasive melanoma cells and (iv) the relative high throughput nature of the system to enable comparison of multiple invasive melanoma lines using the ECIS 96-well plate.

### 4.1. Melanoma Cell-Mediated Effects on the Barrier Are Often Rapid – Importance of Real-Time Data

As described previously, the ECIS system allows assessment of endothelial barrier integrity in real time. This was particularly important as most of the melanoma lines affected barrier integrity within 1 h of their addition to the endothelial apical face, where the barrier resistance started to decline almost instantly. This exemplifies the importance of real-time and automated recording of barrier disruption. Manual recording techniques such as Epithelial Volt/Ohm Meter (EVOM) lack the temporal resolution [11] required to assess such rapid or transient effects on barrier integrity. Time-lapse images were captured using a Nikon BioStation for visual evidence of endothelial barrier disruption. These showed evidence of integration within the endothelium within 60 min, by which time the melanoma cells had created visible gaps (evident by the loss of green stain) in the endothelial layer and had intercalated between the endothelial cells. The disruptive effects observed by microscopy were delayed slightly relative to those seen with the acute ECIS measurements. This can be explained by the fact that visual gap formation is preceded by extensive changes in the molecular integrity of the junctions, as measured sensitively by the ECIS technology.

### 4.2. Melanoma Cell-Mediated Barrier Effects Are Cell Number-Dependent – Importance of ECIS Sensitivity 

Due to the high sensitivity of the ECIS system, changes in resistance could be detected with low numbers of melanoma cell addition, more representative of conditions prevailing in vivo [12]. The initial panel of melanoma lines (NZM7, NZM48 and NZM74) was assessed at a range of ET ratios to establish the sensitivity of the system to detect robust effects of the melanoma cells on the barrier integrity. For each melanoma line, the greatest effect on barrier disruption was seen at an ET ratio of 1:1. For these three melanoma lines, barrier disruption was also observed at an ET ratio of 1:10 (1 melanoma cell per 10 endothelial cells) but to a lesser degree. ECIS did not detect barrier disruption at an ET ratio of 1:100. Therefore, it was evident that the melanoma-mediated barrier disruption was clearly cell number-dependent. This is a crucial observation because previous studies [5,6,7] have often used a single ET ratio to assess melanoma invasion at much higher ratios. These results demonstrate that (i) each melanoma line could disrupt brain-derived endothelium according to our in vitro model, (ii) the disruption was surprisingly rapid and sustained and (iii) similar disruption was not seen upon addition of human PBMCs at the same ET ratios.

### 4.3. Melanoma Cell-Mediated Effect Is Variable – Importance of ECIS Modelling

The primary strength of the ECIS technology comes from the mathematical modelling of the data as established by Giaever and Keese, 1991 [10]. As described previously, the total barrier resistance is comprised of the resistance at the paracellular junctions and the resistance basolaterally due to focal adhesions. The ECIS software can resolve the total resistance into these components to get an estimation of which compartment is changing and when. This is particularly important and unique to the ECIS technology. Our results show that the profile of loss of paracellular barrier resistance replicated almost exactly that of the overall resistance, suggesting that the barrier disruption is mostly attributed to the paracellular junctions. For the NZM7, NZM48 and NZM74 melanoma lines, there was also a distinct effect on basolateral adhesion. Crucially, the changes observed in resistance alpha were consistently later than those observed in Rb. This suggests that Rb decreased first, indicating an opening of the paracellular barrier first, and the basolateral adhesion (Alpha) was affected several hours later. This relationship is fully consistent with the hypothesis that melanoma cell invasion occurs in a paracellular manner. Major changes in Alpha were not detected with the melanoma lines comprising the second panel (NZM 20, 41, 60, 92 and 98), each of which had an effect on the total resistance and the modelled paracellular resistance (Rb). Variations in Alpha resistance reflect variations in the basolateral adhesion of the cells, which likely arise because the endothelial morphology changes and is distorted by invading melanoma cells forcing their way between and through the endothelial cells. Therefore, the lack of effect on Alpha suggests that although some melanoma lines can alter the paracellular barrier integrity, they may lack the capacity to fully open the endothelial cleft and move through for complete invasion. This suggests that different invasive mechanisms may be used by different melanoma lines giving them different metastatic potential. In theory, this could then be attributed to a variety of different molecular factors involved in the invasive nature of the melanoma cells. This is an important aspect revealed by ECIS which requires future in-depth investigation.

### 4.4. Factors Secreted by the Melanoma Cells also Effect the Endothelial Barrier

In addition to the direct cell-mediated effects, we also showed that melanoma-conditioned medium (CM), collected after seven days of culture, decreased endothelial barrier resistance. However, this decrease was less than what was seen with the melanoma cells. Notably, NZM7 CM mediated the greatest decrease in barrier resistance, and for all cell lines the paracellular resistance was affected more than the basolateral. Overall, the CM data reflected similar results to those seen with the melanoma cells, but to a lesser extent. Intriguingly, the temporal profiles of barrier disruption from CM of all cell types were the same—wherein NZM7 did not show faster disruption compared to other cell lines. These results suggested that a combination of mediators might be present for melanoma migration (Figure 9), on the cell surface as adhesion proteins and receptors, or intrinsically secreted by the cells over time.

### 4.5. ECIS Data Predict a Dominant Effect of the Melanoma Cells on the Paracellular Barrier

The key interpretation of the ECIS data from all the melanoma lines assessed in this study is that the melanoma cells primarily target the paracellular junctions and this causes changes in Rb first and dominantly over Alpha (basolateral adhesion). Such large changes in total barrier integrity could result from the melanoma cells simply killing the endothelial cells, causing total loss of the barrier. The modelled ECIS data and the time-lapse imaging data do not support melanoma-mediated killing as the primary mechanism of invasion. We did see evidence of a few endothelial cells dying during the course of some experiments, but these were relatively infrequent. The modelled ECIS data predicted that the melanoma cells targeted the paracellular junctions, and this was supported by the imaging data, which revealed clear and distinct localisation of the melanoma cells at the paracellular space (see Figure 9, Step 1). We suggest the following sequence of events, where melanoma cells first extend protrusions between the endothelium and secrete a variety of molecules to disrupt the paracellular junctions that form the endothelial monolayer (Figure 9, Step 2). The separation of neighbouring endothelial cells enables integration by the proximal melanoma cell to then also disrupt basolateral endothelial integrity and allow melanoma penetration (Figure 9, Step 3).

We conclude from this study that ECIS technology represents a powerful tool for the investigation of melanoma invasiveness. The important aspects of the technology that are valuable are the (i) autonomous nature of data collection, (ii) the high sensitivity of the multi-electrode arrays enabling a lower number of effector cells to be used, (iii) 96-well array systems allowing numerous melanoma lines to be compared or variable culture conditions to be used, including pharmacological blockade, and (iv) the ability to model the total resistance data into Rb and Alpha. In our opinion, this unique aspect of ECIS is very powerful as it can discriminate between invasion as occurring via cell death, paracellular pathways or trans-cellular pathways. In this study, our data strongly support mechanisms of invasion primarily via the paracellular route with a minor component of barrier loss via endothelial cell death.

This paper therefore highlights how the sensitivity of ECIS allows us to learn of the migratory nature of these melanoma cells on our endothelial cell model (hCMVECs). Although numerous papers show evidence of melanoma-mediated endothelial disruption, our ECIS results specify that loss of barrier integrity occurs first at the paracellular site and extends to the basolateral compartment supporting the understanding that migration through the paracellular route requires very specific melanoma cell interaction with the endothelial cell, in a subsequent manner. This suggests a complex migratory mechanism, which cannot be attributed to loss or disruption of junctional proteins alone. Furthermore, the ECIS sensitivity and size allow us to study this across multiple cell-number titrations and multiple cell lines at the same time to compare and assess the invasive capabilities of human melanoma cell lines.

## 5. Conclusions

Highly invasive cancers, including melanoma, that metastasize to the brain have poor clinical outcomes. In individuals with brain metastases, response to current therapies is often reduced and the brain metastases are a major determinant in treatment failure. It is therefore essential that we identify the molecular mechanisms used by invasive cancer cells to successfully breach endothelial barriers of the brain and other tissues. The endothelium represents the interface where potential molecular targets exist by which these interactions could be blocked and invasion prevented. As the ECIS system provides a robust and multimodal method for assessment of endothelial barrier strength, it is invaluable for measuring cancer-mediated disruption of the endothelium and has great potential in future drug discovery studies aimed at discovering the key molecules involved in the invasion of these devastating cells.

## Supplementary Materials

The following are available online at https://www.mdpi.com/2079-6374/9/2/56/s1, Video S1: AVI generated from time-lapse imaging of melanoma cells (NZM7) added to fully confluent layer of hCMVECs. The melanoma cells were added on frame 16 and appear as bright spherical cells. This equates to T = 0 in Figure 7. Each frame is 5 min apart. Imaging conducted using Nikon BioStation (V2.23) and AVI generated using BioStation IM software, Video S2: The endothelial cells are counterstained green and the melanoma cells (NZM74) are added on frame 25. The melanoma cells are phase bright and spherical in appearance. As the melanoma cells invade, the green endothelial monolayer is visibly parted and several gaps appear between neighbouring endothelia. The AVI was created from the BioStation images using the BioStation IM software (V2.23).

## Appendix A

**Table A1:** Materials for cell culture.

Reagent	Company	Catalogue Number
Collagen I – rat tail	Gibco	A1048301
M199	Gibco	11150-067
FBS	Sigma-Aldrich	12203C-500ML
Hydrocortisone	Sigma-Aldrich	H0888
hFGF	PeproTech	PTAF10018B50
hEGF	PeproTech	PTAF10015100
Heparin	Sigma-Aldrich	H-3393
GlutaMAX	Gibco	305050-061
dibutyryl-cAMP	Sigma-Aldrich	D0627
aMEM	Gibco	12561072
Insulin-Transferrin-Sodium Selenite	Sigma-Aldrich	11074547001
RPMI 1640 media	Gibco	11875093

**Table A2:** List of human-derived New Zealand Melanoma (NZM) cell types.

Panel	New Zealand Melanoma Cell line	Research Resource ID
1	NZM48	CVCL_S423
1	NZM7 or NZM7A	CVCL_D843
1	NZM74	CVCL_0D38
2	NZM20	CVCL_D824
2	NZM41	CVCL_S426
2	NZM60	CVCL_S416
2	NZM92	CVCL_0D52
2	NZM98	CVCL_0D58
